# Green turtle tracking leads the discovery of seagrass blue carbon resources

**DOI:** 10.1098/rspb.2024.0502

**Published:** 2024-11-27

**Authors:** Hugo F. Mann, Natalie E. Wildermann, Chuancheng Fu, Hector Barrios-Garrido, Takahiro Shimada, Naira Pluma, Carlos M. Duarte

**Affiliations:** ^1^ Marine Science Program, Biological and Environmental Science and Engineering Division (BESE), King Abdullah University of Science and Technology (KAUST), Thuwal, Makkah 23955-6900, Saudi Arabia; ^2^ Queensland Government, Brisbane, Queensland, Australia

**Keywords:** blue carbon, green turtle, seagrass mapping, satellite telemetry, remote sensing, coastal ecosystems

## Abstract

Seagrass meadows are natural carbon sinks, and their conservation and restoration play a crucial role in climate change mitigation and adaptation. However, blue carbon projects are hindered, in most nations, by major gaps in understanding the distribution and extent of seagrasses. Here, we show how satellite tracking of green turtles (*Chelonia mydas*) provided a major advance in identifying novel seagrass blue carbon resources in the Red Sea. By tracking 53 nesting green turtles, we identified 38 distinctive foraging sites. All ground-truthed foraging sites (100%) identified a seagrass meadow, surpassing the 40% (*n* = 30) accuracy of satellite imagery-based inferences. Sampling from these turtle-derived locations represents a greater range of depths than previously sampled in the Red Sea providing a carbon stock estimate of 4.89 ± 0.83 kg *C*_org_ (organic carbon) m^−2^. By improving estimates of seagrass extent and associated blue carbon, our approach can support the conservation of blue carbon resources in data-deficient regions worldwide.

## 1. Introduction

Blue carbon strategies aim to mitigate and adapt to climate change by preserving and restoring coastal ecosystems that act as intense carbon sinks, such as seagrass meadows [1,2]. These strategies also align with the goals of the Kunming–Montreal Global Biodiversity Framework, which seeks to halt biodiversity losses and restore 30% of degraded habitats by 2030 [3]. However, effectively implementing these strategies requires an inventory of existing and lost blue carbon resources.

While inventories for mangrove forests [4] and saltmarshes [2], which can be easily assessed from space, are relatively well-constrained, seagrass meadows remain poorly mapped. A recent assessment identified 160 387 km^2^ of verified seagrass meadows globally, with a low confidence estimate of up to 266 562 km^2^ [5]. In contrast, niche models project the suitable ocean space for seagrass meadows to be as vast as 1 646 788 km^2^ [6], suggesting the global seagrass area may be much larger than mapped. This uncertainty in seagrass areas and status hampers blue carbon and conservation efforts in many regions.

The main reason for this knowledge gap in global seagrass distribution is the difficulty in resolving seagrass from other marine components using remote sensing. Optical sensing via satellites or airborne platforms struggles to differentiate seagrass from organisms with similar optical signatures, such as green algae [7,8]. In carbonate-rich tropical waters, where most seagrass areas are found, short seagrass canopies tend to accumulate carbonate particles on leaves, which scatters light and obscures seagrass’s optical signature [9]. Consequently, remote sensing-based seagrass mapping is reliable mostly in shallow (<8 m) segments of meadows.

Recent efforts to combine mapping methods facilitated the discovery of the world’s largest seagrass ecosystem (up to 92 000 km^2^) in the Bahamas Bank, using a combination of remote sensing, *in situ* surveys, and tracking of tiger sharks (*Galeocerdo cuvier*) [10]. This discovery substantiates previous suggestions that tracking animals with strong fidelity to seagrass meadows could reveal unknown meadows [11], as demonstrated by the serendipitous discovery of a deep oceanic seagrass meadow in the Indian Ocean through tracking green turtles (*Chelonia mydas*) [12]. They have a high fidelity to a given foraging site, even to the extent of long migrations, which also means that identifying these is important for their conservation [13,14].

Unlike tiger sharks, which are depleted in many regions and challenging to tag, green turtles have expanding populations in some places, and vast ranges [15], crossing exclusive economic zones (EEZs) [16], while usually showing high fidelity to specific food sources [17] such as seagrass and macroalgae [18,19]. These herbivorous turtles can be tagged with satellite transmitters on nesting beaches and tracked to their foraging grounds. However, while individual cases have showcased the potential of tracking green turtles to discover new seagrass meadows [12], systematic regional-scale applications remain unexplored.

We assess the reliability of green turtle tracking in identifying seagrass meadows and associated blue carbon resources in the data-deficient Red Sea and compare the accuracy of green turtles in guiding the discovery of seagrass meadows to inferences from advanced remote sensing systems. Seagrass meadows in the Red Sea can grow in deep waters (up to 70 m) [17], but consistent mapping efforts are lacking, with limited data primarily from Egypt and Saudi Arabia [20]. Several nations in the Red Sea region, such as Eritrea, Somalia, Djibouti, Yemen and Sudan, face research constraints due to economic challenges and social unrest. Hence, green turtles can play a crucial role in mapping seagrass meadows, especially in the Red Sea where their populations are increasing [21] as they freely traverse EEZs, making them valuable partners for expanding our understanding of seagrass distribution and associated blue carbon resources in the Red Sea, which can then be protected and, if needed, restored, thereby benefiting green turtles as well.

## 2. Results

### (a) Foraging sites

Thirty-eight foraging sites were identified (electronic supplementary material, figures S1–S32) from the satellite telemetry data from 53 tagged green turtles (electronic supplementary material, table S2), with multiple green turtle individuals sharing the same foraging sites. The majority of the green turtle individuals tracked (*n* = 53) foraged in the northern part of the Red Sea (North of the midpoint 20.25˚N), possibly reflecting the northern positions of the nesting beaches where turtles were tagged, but some individuals also foraged along the coast of every country bordering the Red Sea except Djibouti, as well as in the Gulf of Suez and Gulf of Aqaba (figure 1*a*; electronic supplementary material, figure S33). The turtles travelled 4.48 to 1275.31 km from the nesting beaches where they were tagged to the foraging sites, with a median distance of 360.64 km (figure 2*a*).

**Figure 1.**
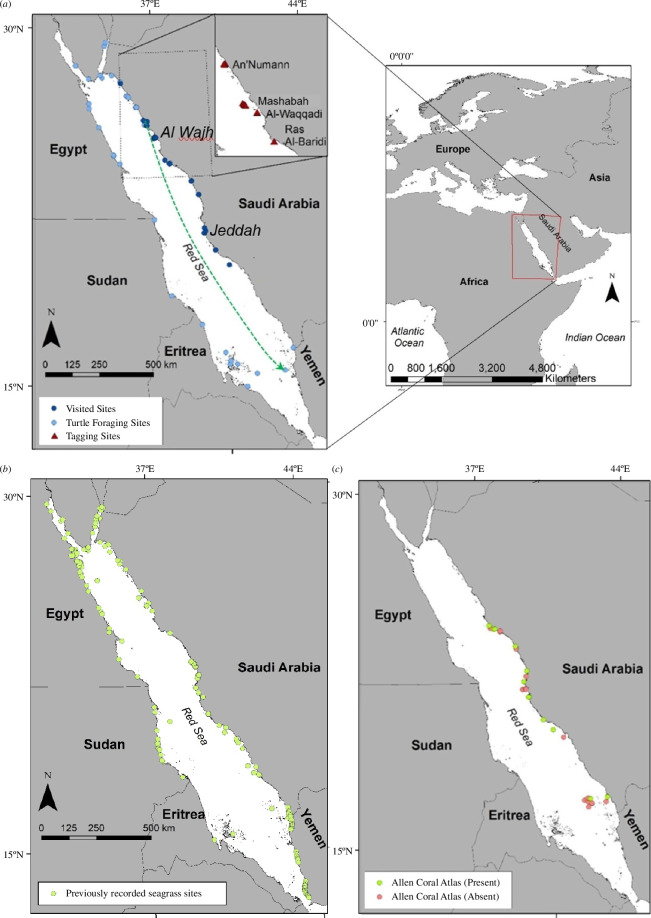
Spatial distribution of green turtle foraging, seagrass and sampling sites. (*a*) Foraging sites identified by green turtle satellite telemetry (light blue) and foraging sites that were ground-truthed and sampled for carbon stocks (dark blue); the green arrow shows the longest distance travelled by a turtle. (*b*) Seagrass meadows reported in previous studies (see electronic supplementary material, table S1) and (*c*) seagrass meadows resolved from the Allen Coral Atlas in the Red Sea, which were ground-truthed indicating where seagrass was present (green) and absent (pink).

**Figure 2.**
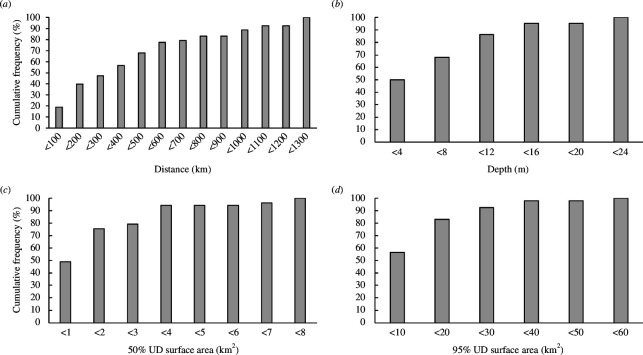
Frequency distribution of different metrics derived from green turtle satellite tracking in the Red Sea. (*a*) Cumulative frequency of distances travelled from the nesting site where the turtle was tagged to foraging sites (*n* = 53). (*b*) Cumulative frequency of depths at the foraging sites visited and ground-truthed (*n* = 14). (*c*) Cumulative frequency of the surface areas of the 50% utilization distributions (UDs) of the foraging sites (*n* = 53). (*d*) Cumulative frequency of the surface areas of the 95% UDs of the foraging sites (*n* = 53).

**Figure 3.**
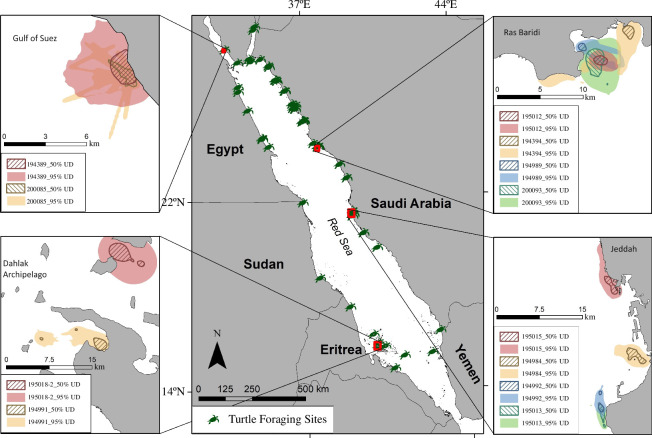
Utilization distributions (UDs) of some green turtles foraging in the Gulf of Suez (Egypt), Dahlak Archipelago (Eritrea), Ras Baridi (Saudi Arabia) and Jeddah (Saudi Arabia). 95% UDs (solid transparent colours) and 50% UDs (line fill) are shown for 12 green turtles at four different locations. The rest of the UD maps are available in electronic supplementary material, figures S1–S32. Foraging sites are represented by green turtle icons.

Ground-truthed foraging sites ranged broadly in depth (figure 2*b*), from 0.5 to 22.2 m, with a mean (± s.e.) depth of 5.77 ± 1.20 m, with the shallowest and deepest sites off Al Wajh lagoon (electronic supplementary material, figures S11 and S12, respectively), and a similarly shallow site (0.5 m) off the city of Jeddah (electronic supplementary material, figure S18; figure 3). Fifteen of the 22 locations retrieved from green turtle telemetry data were less than 8 m deep, while seven foraging sites were deeper than 8 m (figure 2). The majority of the 50% utilization distributions (UDs), which represent the core area used by foraging turtles, had a surface area of less than 2 km^2^, with a mean ± s.e. of 3.00 ± 0.55 km^2^ and a median area of 1.48 km^2^ (figure 2*c*). The mean ± s.e. size of the 95% UDs, which more broadly represent the size of each foraging ground, was 17.42 ± 2.83 km^2^ with a median area of 9.98 km^2^ (figure 2*d*).

### (b) Discovery of unknown seagrass meadows

We compiled a data set with the position of seagrass meadows in the Red Sea reported from the published literature [20,22–53] (figure 1*b*; electronic supplementary material, table S1). Most of these were distributed in the northern Red Sea, particularly in Saudi Arabian and Egyptian waters, possibly reflecting the distribution of research. There was also a large number of seagrass meadows recorded in Yemen. Eight of these 263 previously recorded seagrass sites overlapped only with four of the seagrass meadows identified by the tagged green turtles using 95% UDs. Hence, 34 of the 38 distinctive sites identified by the tracked green turtles, or 89.47% of the total sites recorded in this study, represent updated records of seagrass meadows for the Red Sea. Based on this, it can be inferred that green turtles discovered 34 new seagrass sites, extending the total number of reported seagrass meadows in the Red Sea by 12.93%, relative to those extracted from the literature.

We visited 22 sampling sites indicated by green turtles, and as some of these were part of the same extensive seagrass meadow, this represented a total of 14 ground-truthed seagrass meadows (figure 1*a*; table 1). The green turtles were 100% successful in discovering seagrass meadows at the 14 ground-truthed sites. In contrast, we found seagrass only at 12 of the 30 Allen Coral Atlas-derived sites (figure 1*c*), which represents a 40% accuracy. The number of sites where the Allen Coral Atlas and green turtle predicted seagrass was used to generate expected values for the number of sites where seagrass would be present or not present, assuming that the probability of green turtles and the Allen Coral Atlas was the same.

**Table 1.** Seagrass presence or absence in the Allen Coral Atlas for ground-truthed meadows identified in the Red Sea from green turtle satellite tracking. Where multiple depths for the same meadow were taken, they are shown as mean (range).

seagrass meadow	turtle ID	latitude	longitude	present in Allen Coral Atlas	depth (m)
1	194397	20.594	39.567	No	0.7
2	195013, 194992	21.327	39.097	No	0.5
3	194985	19.962	40.146	Yes	1.5
4	195004	23.511	38.607	Yes	1.6
5	200093, 195012, 194989	24.260	37.648	Yes	1.5
6	195018-1	25.355	36.994	No	6.9
7	194984	21.406	39.151	No	1
8	195015	21.534	39.114	No	1
9	200086, 194986	22.929	38.906	No	12.5
10	195000	25.384	37.005	No	6
11	194988	25.372	37.051	No	12
12	200101, 194398	26.049	36.677	No	5.5 (1–10)
13	194398, 200100-1, 200100-2	26.103	36.507	No	10.9 (0.5–22.2)
14	200100-3	25.881	36.653	No	6.8

Our results show that there was a significant difference between the accurate detection of seagrass between green-turtle tracking and the advanced remote sensing used by the Allen Coral Atlas (*p* < 0.00058, d.f. = 1, *χ^2^* = 11.842; table 2). Overall, the turtles were 18.5 times more likely than the Allen Coral Atlas to correctly identify seagrass meadows (see methods for odds ratio analysis). Only 3 out of 14 ground-truthed turtle-derived foraging sites were also identified as seagrass meadows by the Allen Coral Atlas (table 1), or 21.43%, indicating the presence of many false negatives in the Allen Coral Atlas.

**Table 2.** Contingency table comparing the reliability of green turtles and the satellite imagery using Allen Coral Atlas to identify seagrass meadows. Numbers represent observed values with expected values shown as subscripts.

	green turtles	Allen Coral Atlas
seagrass present	14_8.27_	12_17.73_
seagrass absent	0_5.73_	18_12.27_
*p*‐value	0.00058	
*X* ^2^	11.842	

### (c) Sediment properties and blue carbon stocks

The mean dry bulk density (DBD) for the top sediment layer in the seagrass meadows found as foraging sites for tagged green turtles sampled was 1.57 ± 0.049 (±s.e.) g cm^−3^, with a range from 0.81 to 2.63 g cm^−3^ (table 3). The mean DBD was lower for the middle section, 1.43 ± 0.020 (±s.e.) g cm^−3^, and the range was 1.11–1.78 g cm^−3^. The bottom mean DBD was higher than the middle but lower than the top, at 1.50 ± 0.030 (±s.e.) g cm^−3^, with a range of 1.15–3.07 g cm^−3^. The 0–1.0 cm sediment layer had a mean C_org_ concentration of 0.36 ± 0.026 (±s.e.) % (range 0.069%–1.45%), comparable to that in the 1.0–5.0 cm layer, with a mean content of 0.32 ± 0.020 (±s.e.) % (range 0.05%–0.86%), and the bottom layer, with a mean value of 0.34 ± 0.021 (±s.e.) % (range 0.049%–1.00%).

**Table 3.** Sediment DBD and organic carbon estimates for each sediment depth layer.

depth (cm)	count	mean C_org_ (%)	range C_org_ (%)	s.d. C_org_ (%)	mean DBD (g cm^−3^)	range DBD (g cm^−3^)	s.d. DBD (g cm^−3^)
0.0–1.0	66	0.36	0.069–1.45	0.21	1.57	0.81–2.63	0.40
1.0–5.0	65	0.32	0.05–0.86	0.16	1.43	1.11–1.78	0.16
5.0–10.0	66	0.34	0.049–1.00	0.17	1.50	1.15–3.07	0.25

The mean (±s.e.) sediment carbon stock of the seagrass meadows identified through green turtle telemetry, extrapolated to a sediment depth of 1 m to allow comparison with published estimates, was 4.89 ± 0.83 kg C_org_ m^−2^, ranging from 1.41 kg C_org_ m^−2^ to 8.87 kg C_org_ m^−2^ (median 5.15 kg C_org_ m^−2^). Extrapolating this mean value to the surface area of the 95% UD (850.61 km^2^) of all foraging sites identified in this study allows a first estimate for seagrass blue carbon resources found by green turtles of 4.16 ± 0.71 (95% CI) Tg C_org_. Extrapolating the mean to the 50%UD (156.04 km^2^) gives us a more conservative estimate of 0.76 ± 0.13 Tg C_org_.

## 3. Discussion

Our results demonstrate that adult female green turtles can serve as indicators of the location of seagrass meadows, guiding the identification of 34 hitherto unknown seagrass meadows in the Red Sea. This finding increased the recorded distribution of seagrass meadows in the region by nearly 13%. While these meadows were largely distributed in the north of the Red Sea, potentially due to the northerly situation of the nesting beaches and preference for relatively shorter migration distances (figure 2*a*), foraging sites were found throughout the Red Sea. Furthermore, since the 53 green turtles were tracked from only four nesting beaches in the northeastern Red Sea, it is reasonable to assume that expanding tracking efforts to other regions of the Red Sea will likely uncover additional meadows. Since the tracked turtles were present on the coast of every country that borders the Red Sea except Djibouti, this highlights another advantage of using green turtles to identify seagrass meadows in the EEZ of nations with limited marine research capabilities due to economic constraints and/or social unrest.

The fact that even in the northern Red Sea, which has been extensively surveyed, almost 9 out of every 10 seagrass meadows identified by green turtles were novel, suggests that we are still far from having a full inventory of seagrass meadows in the Red Sea, where the reported seagrass area remains grossly underestimated. Increasing tagging sites throughout the coast of the Red Sea, particularly in the South will most likely help to discover additional seagrass meadows, and increase the coverage estimates of known seagrass meadows in this poorly studied sea.

The high reliability of the identification of seagrass by green turtles combined with the known issues with remote sensing (i.e. depth limitation [54] and optical overlap with other habitat types [8]) highlights the capacity of employing adult green turtles to identify seagrass meadows in understudied regions, which include almost all developing coastal nations. We make the assumption in this study that all of the green turtle foraging sites are seagrass meadows based on the 100% seagrass presence at the ground-truthed foraging sites. This is supported by regional studies that have found seagrass to be the majority contributor to adult green turtle diet. For example, in the West Indian Ocean, adult non-gravid female green turtles were found to have 95% seagrass in their stomach contents [17].

While the selectivity of green turtles when feeding on seagrass benefits us in identifying seagrass meadows, they are also selective to the extent where they exclude certain species of seagrass that are not easy to digest. Hence, monospecific meadows of these non-palatable seagrass species, such as *Enhalus acoroides,* may not be represented among green turtles-derived locations. Indeed, high pressure on target seagrass species may lead to shifts in seagrass species composition, and, where green turtle populations increase and their natural predators, tiger sharks, are depleted, there is a risk of overgrazing leading to the collapse of seagrass meadows altogether [55].

When combined, the total estimated area of seagrass meadows in the Red Sea reaches 850.61 km^2^ (95% UD) which is more than a twofold increase from the only previously reported area estimate for overall seagrass cover here (370 km^2^ [22]). Combining published (370 km^2^ [22]) and new area estimates (850.61 km^2^, adopting 95% UD) results in a maximum potential area of seagrass meadows in the Red Sea of 1120.61 km^2^, assuming no overlap between the two estimates. In comparison, there are 175.3 ± 0.93 km^2^ of mangroves [56], and 13 605 km^2^ of coral reef habitat [57] in the Red Sea. The UDs derived from green turtles' telemetry provide a first-order estimate for the surface area of a seagrass meadow. Nevertheless, it is important to account for the influence of other behaviour-moulding pressures, in particular predation [15,58–61], which are likely to increase the apparent surface area used by green turtles at the identified foraging sites making this an unlikely method to accurately gauge the surface area of the meadows. For example, higher predation could lead to the foraging area constricting, so the whole seagrass meadow would not be used for foraging.

Remarkably, green turtles were far superior to advanced remote sensing techniques which combine high-resolution satellites, 3 m resolution and AI-assisted classification in identifying seagrass meadows (table 2). All of the ground-truthed foraging sites identified by green turtles were seagrass meadows, whereas only 40% of habitats identified by the Allen Coral Atlas as seagrass meadows were confirmed as seagrass meadows. Moreover, 78.57% of the seagrass meadows discovered by tracking green turtles were not reported as seagrass meadows by the Allen Coral Atlas, which limits its information to habitats shallower than 5 m, a depth exceeded by 57.14% of the turtle foraging sites. Green turtles derived locations identified deeper meadows leading to a more representative understanding of the seagrass meadow distribution in the Red Sea than could be obtained only through remote sensing. Approximately 1/3 of the sites discovered were deeper than 8 m, probably beyond the detection capacity of aerial and satellite images [5]. Even accounting for the fact that the depth to which these methods' detection capacity may be extended in oligotrophic waters, this still implies the existence of significant areas of seagrass that cannot presently be identified and mapped by satellite images.

Green turtles lead to the identification of seagrass meadows from a wider range of depths (0.5–22 m) than had previously been sampled for carbon stock estimates in the Red Sea (0.5–8 m) [22,23]. As the carbon storing capacity of seagrass varies with depth [62], these areas need to be accounted for in a representative estimate of seagrass carbon stocks. The carbon stocks estimated in this study (4.89 ± 0.89 kg C_org_ m^−2^; table 4) were lower than those estimated by Garcias-Bonet *et al*. (7.19 ± 0.38 kg C_org_ m^−2^) [63] when integrated into 1 m depth in the sediment, possibly as a result of the deeper sites included in our assessment [62]. However, our estimates were higher than those reported by Serrano *et al.* (3.4 ± 0.3 kg C_org_ m^−2^) [22] for seagrass meadows in the Central Red Sea. Hence, our study likely provides a more representative estimate of carbon stocks in the Red Sea. We provide a conservative estimate of carbon stored in seagrass meadows discovered by green turtles based on the 50% UDs of the foraging areas integrated to 1 m depth of sediment of 0.76 ± 0.13 (±95% CI) Tg C_org_, and an upper limit, based on the 95% UDs, of 4.16 ± 0.71 Tg C_org_. Furthermore, there are 255 known sites from previous studies that were not visited by the subset of green turtles tracked in this study. The organic carbon stocks in Red Sea seagrass meadows identified by green turtles are about half of the global average [65], but similar to carbon stocks in seagrass meadows in Abu Dhabi and study by Lavery *et al.* [40] in Australia which included seagrass meadows from different climatic regions (table 4). The carbon stock estimates were extrapolated to represent 1 m deep sediment, which is a standard based on REDD+protocol [70], thereby allowing the stock estimates to be compared to previous estimates (table 4). However, as there is little evidence of a drop off of carbon concentration with depth in seagrass sediment [71], and since in some of the sampled sites, the sediment was not even 10 cm deep, this standard needs to be revised in the future to shallower sediment depths to more accurately reflect the carbon stored in seagrass meadows.

**Table 4.** Comparison between carbon stock estimates from this study and other published studies on seagrass, integrated to 1 m for consistency. *This value was converted from Mg C ha− 1 for only the top 5 cm of sediment.

region	cores (*n*)	carbon stock (kg C_org_ m^−2^)	study
Red Sea	66	4.89 ± 0.89	This study
Red Sea	21–28	7.20 ± 0.4	[63]
Red Sea	27	3.4 ± 0.3	[64]
Global	—	11.15 ± 0.4	[65]
Abu Dhabi	40	4.91 ± 0.7	[66]
Bahamas	21	7 ± 2	[10]
Asia	21	7.24 ± 2.2	[67]
Australia	28+previous	12.8 ± 0.7	[52]
China		14.04 ± 7.14*	[68]
Australia		5.05 ± 0.83	[69]

In summary, our results highlight the potential and robustness of tracking green turtles to their foraging sites to discover and map seagrass ecosystems and their associated blue carbon resources in the Red Sea [11]. The growing abundance in some regions, large biogeographic range and migration of green turtles identify them as a valuable tool in mapping seagrass meadows across the tropical and subtropical region, where seagrass areas remain poorly documented. This is important to calculate the seagrass blue carbon resources of coastal developing nations, which conservation and adaptation may contribute to mitigate and adapt to climate change. Identifying seagrass meadows enables their conservation as regulations can be put in place to protect them, and consequentially protecting the essential services they provide to the environment and society. This is essential, in turn, to support the goals of the Kunming-Montreal Global Biodiversity Framework, which calls for protecting 30% of ocean space, where seagrass meadows are underrepresented, and restoring 30% of degraded terrestrial, inland waters, coastal and marine ecosystems [3]. It can also help formulate blue carbon projects, thereby contributing to climate action and delivering benefits to the nations, as economic benefits in the form of carbon credits can be attached to blue carbon resources, as well as inform the contribution of seagrass ecosystems to other vital services that it provides, as coastal protection [1] and supporting fisheries [72,73].

## 4. Methods

### (a) Turtle sampling

Fifty-three nesting female green turtles were tagged with SPLASH 10 Argos-linked FastLoc GPS satellite tags (Wildlife Computers), which transmit location data to satellites when the turtle surfaces to breathe. One was tagged in 2018, 28 in 2019 and 24 in 2021 at An’Numann (*n* = 4), Mashabah (*n* = 36) and Al-Waqqadi (*n* = 3) Islands in the Red Sea Global (RSG) and Ras Al Baridi (*n* = 10). They were tagged between July and December using the protocol described by Shimada *et al.* [74]. Per standard practice, the tagging was performed after the females had finished nesting to prevent negative impacts on their reproductive success.

We gathered the FastLoc GPS locations derived from the satellite telemetry data from each turtle for up to a year (mean tracking duration 210.94 days, range 1432.37 km). The foraging site for each turtle was identified by projecting the track on Google Earth. Areas where the tortuosity of the track increased, and it crossed over itself were identified as foraging sites following similar principles as [75] and [76] and visually assessing where the angle of advance decreased and crossed over itself multiple times (electronic supplementary material, figures S34 and S35). Both conditions were required for identification of a foraging site, as a mere increase in track tortuosity would likely indicate a search pattern. The GPS locations within each foraging site were isolated, and then locations derived from less than 5 satellites and with a residual value higher than 35 were removed [77]. It was assumed that green turtles were only foraging during the day and resting at night [78], so the nighttime data was removed in R using the ‘suncalc’ package and correcting for Greenwich Mean Time (GMT). The data were then filtered using the package ‘SDLfilter’ and the functions ‘vmax’ and ‘ddfilter’ were used to remove biologically unlikely points [79]. UDs represent the probability that an individual will be distributed in an area in terms of space and time. UDs were estimated using the Biased Random Bridges approach [80] through the ‘BRB’ function within the R package ‘adehabitatHR’ in R v. 4.1.1 [81]. We then computed isopleths representing areas representing 50% (or core areas) and 95% (or home ranges) of the UD density, using the ‘getverticeshr’ function. The surface area (in km^2^) was calculated in ArcMAP 10.8.1 (figure 3; electronic supplementary material, figures S1–S32) and the centroid of the 50% UD polygon was extracted.

### (b) Ancillary data on seagrass distribution and carbon stocks

The reliability of green turtles in identifying seagrass habitats was compared to that of remote sensing. The Allen Coral Atlas was used as a proxy for remote sensing [82]. The Allen Coral Atlas is an online resource that maps benthic habitats, including seagrass, down to 20 m and between 30°N and 30°S in clear waters (Accessible through: https://allencoralatlas.org/). The imagery is obtained at a pixel size of 3.125 m using PlanetScope satellites operating in three visible and four analytical spectral bands and processed for image correction [83]. The Allen Coral Atlas released data collected from August 2018 to September 2020, whereas the green turtle data collected in our study was collected from 2019 to 2022.

In addition, locations of seagrass meadows previously reported in the Red Sea [20,22–53] were extracted from the published literature to investigate the overlap with green turtle-derived sites (95% UD) and determine how many of the seagrass meadows discovered by the turtles were previously unknown.

### (c) Ground-truthing

The centroid of each 50% UD was used to identify sites to ground-truth the presence of seagrass and three random locations in the foraging site were sampled. Each of the 53 turtles reached a foraging site, and as some of the centroids coincided in the same area we recorded a total of 38 distinctive foraging sites. Fourteen of the foraging sites along the Saudi Arabian coast were visited between 5 February 2022 and 4 June 2022, and 22 sampling points (one for each turtle within these 14 foraging sites) were ground-truthed by snorkelling and SCUBA. The depth was taken from the depth of the dive needed to visit the site, which is taken from the deepest point recorded on the dive computer.

A total of 30 sites where the Allen Coral Atlas identified seagrass meadows were also visited and ground-truthed using the same method as above.

### (d) Statistical analyses

Expected values for the presence and absence of seagrass at sites indicated by green turtles and the Allen Coral Atlas were generated in a contingency table using the ‘ctable’ function in R v. 4.3.1. *p-*values comparing the presence and absence of seagrass at sites indicated by green turtles and the Allen Coral Atlas are associated with the *χ*^2^ test which was carried out using the ‘chisq.test’ function. This assessed the probability that green turtles were more accurate at identifying seagrass meadows than the Allen Coral Atlas. An odds ratio was then carried out using the ‘epitools’ package and ‘oddsratio’ function in order to ascertain the difference between likelihoods of the Allen Coral Atlas and green turtles correctly identifying seagrass.

### (e) Carbon content analysis

At each of the 22 green turtle-derived sampling locations visited, three cores were taken throughout the foraging site at least 5 m from each other and, where possible, all relevant seagrass species were present. Plexiglass corers with a diameter of 9.5 cm were used to take 10 cm deep cores, which were then sliced into a top 1 cm, a middle 4 cm and a bottom 5 cm for carbon content analysis.

The core samples were weighed, dried and weighed again to ascertain the DBD. The DBD is used to calculate the sediment mass at different depth intervals, which allows us to calculate the integrated carbon stock. They were then milled to a fine powder using the Retsch PM 200 Mill Grinder, or Planetary Ball Mill at 450 rpm for 3 min. The vessels were cleaned thoroughly in between samples using hydrochloric acid (HCl) and ethanol to prevent cross-contamination. Approximately 15 mg of the sample was weighed into a silver capsule and 20 μl of 3 M HCl was added and the sample was left to dry, then a further 20 μl 3 M HCl was added, and this process repeated until the sample stopped reacting and bubbling to the addition of HCl. This removed the inorganic carbon so that only C_org_ would be measured [84]. After it had stopped reacting, the sample was dried. The silver capsule was then folded and wrapped up in a tin capsule. Then the percentage of C_org_ in each sample was measured with a Thermo Scientific FLASH 2000 CHNS/O, with a furnace temperature of 950℃ and oven temperature of 65℃.

The volume of each core section was calculated from the height and radius. The dry weight was divided by the volume to provide the DBD. The DBD was multiplied by the percentage of C_org_ to determine carbon density, which was averaged across the sections of each core. A mean carbon density with 95% CIs was calculated for each seagrass meadow from three replicated cores taken per site. To extrapolate the amount of carbon stored per seagrass meadow, the carbon density of the top 10 cm was integrated to 1 m depth for comparability with Fourqurean *et al.* [85], Jiang *et al.* [68] and Serrano *et al.* [22], converted into kg m^−2^ of sediment surface area and then applied to the area of the respective seagrass meadow. The mean carbon stock for the 14 sampled foraging sites was applied to the non-sampled foraging sites identified by green turtles and multiplied by their surface area. These were then summed to provide a first estimate for seagrass blue carbon resources found by green turtles.

Organic carbon estimates were also extracted from recent studies of seagrass carbon stocks to compare to the carbon stock data obtained from this study. Seagrass carbon stock estimates were obtained for locations in the Red Sea [64], Abu Dhabi [66], the Bahamas [10], China [68], Asia [67] and Australia [69], and a global estimate was obtained from Kennedy *et al.* [65].

## Data Availability

All processed home range outputs, sediment core data and previously identified seagrass meadows can be accessed through the KAUST Dataset Repository for processed green turtle satellite telemetry, polygons generated in ArcMap of 95% and 50% utilization distributions in foraging sites [86], dry bulk density and organic carbon content from seagrass meadow sediment in the Red Sea [87] and previous seagrass meadow locations [88]. Due to sensitive location data of endangered species, access to the raw sea turtle location data is available upon reasonable request to the corresponding author. Supplementary material is available online [89].
